# Significant response to anti-PD-1 based immunotherapy plus lenvatinib for recurrent intrahepatic cholangiocarcinoma with bone metastasis: A case report and literature review

**DOI:** 10.1097/MD.0000000000017832

**Published:** 2019-11-11

**Authors:** Wei-xun Chen, Gan-xun Li, Zheng-nan Hu, Peng Zhu, Bi-xiang Zhang, Ze-yang Ding

**Affiliations:** Hepatic Surgery Center, Department of Surgery, Tongji Hospital, Tongji Medical College, Huazhong University of Science and Technology, Wuhan, China.

**Keywords:** bone metastasis, cholangiocarcinoma, lenvatinib, nivolumab, PD-1

## Abstract

**Introduction:**

The prognosis for recurrent intrahepatic cholangiocarcinoma with bone metastasis remains dismal and its treatment poses a challenge for oncologists. To date, only 2 cases were reported in which pembrolizumab, an agent against programmed cell death protein-1 (PD-1), combined with chemotherapy led to a complete response.^[1]^ The safety and efficacy of nivolumab-based immunotherapy combined with lenvatinibin intrahepatic cholangiocarcinoma is unknown.

**Patient concerns:**

A 40-year-old female was identified as having a lesion of 7.0 cm in diameter in the right lobe of the liver. In addition, calculi in the main and left hepatic bile ducts as well as the gallbladder were found.

**Diagnosis:**

Based on the results of imaging studies and tumor biomarker level, the patient was initially diagnosed as having intrahepatic cholangiocellular carcinoma and cholelithiasis, after which surgery was performed. The pathological examination confirmed that the tumor was cholangiocarcinoma. Adjuvant chemotherapy was administered after surgery. However, the patient developed recurrent lesions at the 5th month after surgery, and the cholangiocarcinoma expanded to the right thoracic vertebral pedicle (T7–8) at the 6th month.

**Interventions:**

The patient underwent percutaneous microwave ablation after recurrence in the liver was identified. After that, the patient received nivolumab plus lenvatinib.

**Outcomes:**

The lesions in the liver decreased in size and disappeared after treatment with nivolumab plus lenvatinib. Additionally, the metastases in the right thoracic vertebral pedicle were stable after 9 months of therapy.

**Lessons:**

Immunotherapy has revolutionized the treatment of non-small-cell lung cancer, melanoma, and advanced renal cell carcinoma. In this case, the patient achieved an excellent radiological and symptomatic response after receiving nivolumab plus lenvatinib combination therapy. Patients suffering from cholangiocarcinoma with dMMR status and a high tumor mutation burden (TMB) may have a consistent eutherapeutic effect with anti-PD-1-directed treatment.

## 1 Introduction

Cholangiocarcinoma (CCA) is an aggressive cancer of the biliary duct system with extremely poor therapeutic outcomes due to widespread metastasis, high drug resistance, and a lack of effective treatment options.^[2]^ The 5-year overall survival (OS) for CCA following resection is between 21and 35%.^[3–5]^ Liver nitrosamine exposure and fluke infestation are the 2 main exposure risk factors that were identified in patients in northeastern Thailand where CCA is endemic. Possible risk factors for fluke-negative CCA include chronic HBV/HCV virus infection and liver diseases such as biliary calculi, primary sclerosing cholangitis, cirrhosis, and congenital biliary malformations.^[6–9]^ A enhanced understanding of the genetic aberrations that are the main drivers of each disease subtype is integral to establishing a precision medicine approach to cholangiocarcinoma therapy. Identification of biomarkers for the selection of patients harboring pertinent genetic aberrations is an essential precondition for targeted therapy. Nivolumab (Opdivo) is a PD-1-binding IgG4 immunoglobulin that acts as an immune checkpoint inhibitor by selectively blocking the interaction between PD-1 expressed on activated T cells, and its ligands PD-L1 or PD-L2 expressed on immune cells and tumor cells. It has shown activity against a wide spectrum of advanced cancers. In studies of small numbers of cholangiocarcinoma tumor samples (n = 54–99), PD-L1 expression was found on 9% to 72% of specimens, and in 46% to 63% of immune cells within the tumor microenvironment.^[10–12]^ Tumor DNA mismatch repair (MMR) deficiency and/or microsatellite instability (MSI) are examples of genetic aberrations that are associated with high rates and durability of responses to immune-checkpoint inhibitors across some tumor types, including melanoma, NSCLC, and urothelial carcinoma.^[13–15]^ Notably, 5% to 10% of cholangiocarcinomas showed evidence of MMR deficiency.^[16]^ The anti-PD-1 antibody pembrolizumab has been approved by the FDA for the treatment of patients with metastatic or unresectable dMMR and/or MSI-high solid tumors after initial therapy, which would include those with cholangiocarcinoma (https://www.fda.gov/drugs/informationondrugs/approveddrugs/ucm279174.htm). These data predict that PD-1 or PD-L1 inhibitors might provide a new therapeutic option for a substantial proportion of cholangiocarcinoma patients.

Tyrosine-kinase signaling via the fibroblast growth factor receptor (FGFR) and hepatocyte growth factor receptor (MET) is essential for a myriad of cellular processes, including embryogenesis, angiogenesis, tissue homeostasis, wound repair, and cell survival. Several early-phase clinical trials involving patients with advanced-stage cholangiocarcinoma investigated the efficacy of multitargeted tyrosine kinase inhibitors. However, the corresponding phase II studies showed only disappointing effects on progression-free survival (PFS) and overall survival.^[17,18]^ Lenvatinib (E7080) is another multitargeted kinase inhibitor of FGFR1–4, VEGFR1-3, KIT, RET, as well as PDGFR-β.^[19]^ The FDA approved lenvatinib in 2015 for the treatment of progressive, locally recurrent or metastatic, radioactive iodine-refractory differentiated thyroid cancer, or unresectable thyroid cancer.^[20]^ Phase I clinical trials have demonstrated the activity of lenvatinib against multipletypes of cancer, including melanoma, and renal cell carcinoma.^[21]^ The published phase II clinical trials include lenvatinib as monotherapy for unresectable biliary cancer (NCT02579616), a comparison of lenvatinib with everolimus in renal cell carcinoma (NCT02454478), and lenvatinib with sorafenib in hepatocellular carcinoma (NCT01761266).

A detailed search of www.clinicaltrials.gov identified 4 ongoing studies evaluating the efficacy of nivolumab in patients with cholangiocarcinoma, including nivolumab plus entinostat. However, there is no specific information on the efficacy of nivolumab plus lenvatinib in patients with cholangiocarcinoma. Here, we report the case of a 40-year-old Asian woman with recurrent and metastatic cholangiocarcinoma who received second-line nivolumab plus lenvatinib combination therapy. She showed an excellent symptomatic and radiological response to this combination treatment and obtained disease control of bone metastasis after 9 months of therapy. In addition, we reviewed and analyzed the available literature to elucidate the role of immune checkpoint blockade in the treatment of cholangiocarcinoma, as well as discuss the safety and efficacy of nivolumab/lenvatinib in various solid tumors.

## 2 Case report

**Figure 1:**
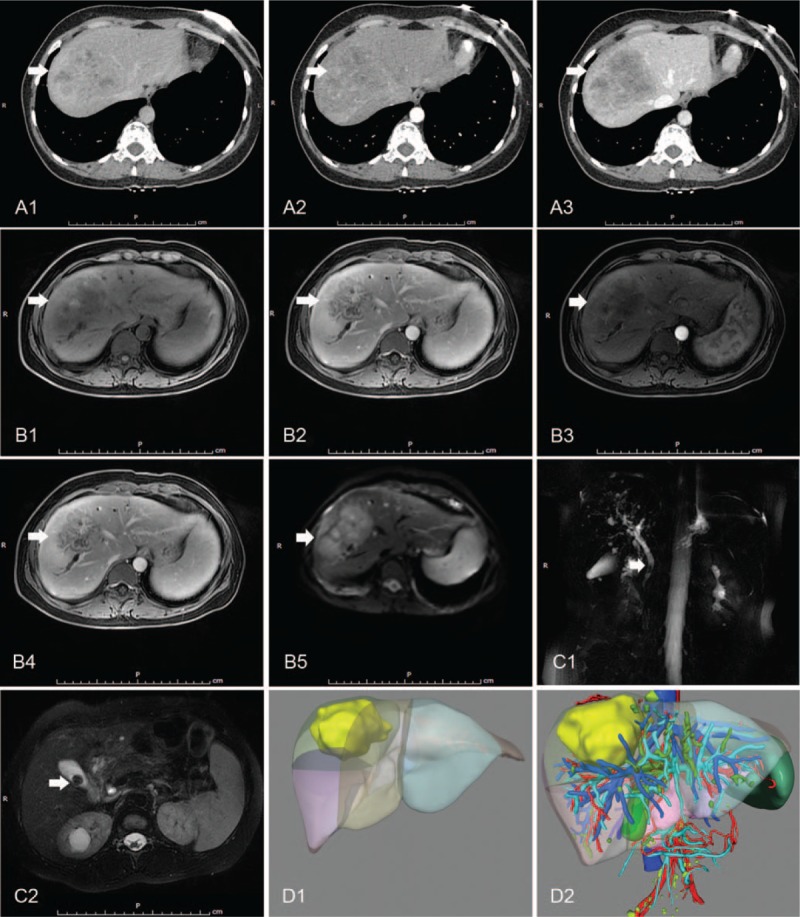
Preoperative radiological examination of the reported case. (A, B) Abdominal computed tomography (CT) scanning and abdominal MRI scanning showed a hypoattenuating liver lesion which located in the segment 8 and was adjacent to right and middle hepatic vein. From (A) to (B), the white arrow heads direct the liver lesion. (C) Magnetic resonance cholangiopancreatography (MRCP) scanning showed calculi were present in the main, left hepatic bile ducts and cholecyst; the white arrow heads direct the calculi. (D) 3D reconstruction of CT images. The liver lesion is labeled with yellow, hepatic artery are labeled with red, portal vein and its branches are labeled with cyan, and hepatic vein and inferior vena cava are labeled with blue.

**Figure 2:**
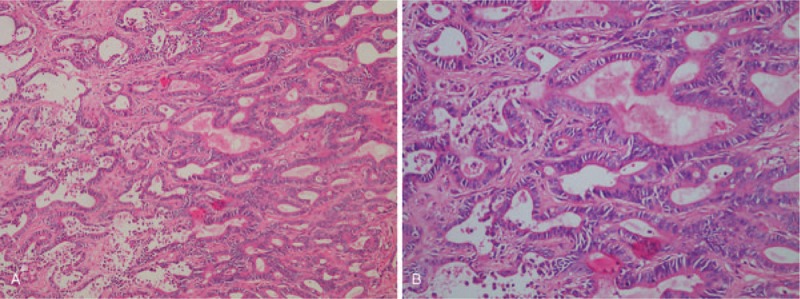
Postoperative pathological examination of the reported case. (A) Hematoxylin and Eosin, Original magnification: 100×; (B) HE Original magnification: 200×.

A 40-year-old female patient was referred to our hospital because of a diagnosis of intrahepatic cholangiocellular carcinoma, which was identified by magnetic resonance imaging (MRI) and computed tomography (CT) during a physical examination. She occasionally felt abdominal pain during 3 days. She did not recall any history of chronic liver disease and reported having undergone caesarean sections in 2007 and 2012. Physical examination was normal except for a previous operation scar. One aunt and 1 elder brother had died due to liver disease. The results of laboratory tests were mostly in the normal range, except for hemoglobin 95.0 g/L (normal range: 115–150 g/L) and tumor biomarkers including carbohydrate antigen 19-9 (CA19-9) 2131.00 U/ml (normal value: <37 U/ml), and carbohydrate antigen 125 (CA125) 134 U/ml (normal value: <35 U/ml). The Child–Pugh grade was A (score 5). Enhancement computed tomography (CT) scanning showed a 8.0 cm × 7.8 cm × 6.2 cm mass in segment VIII adjacent to the right and middle hepatic veins (Fig. 1A1), which was heterogeneously enhanced in the arterial phase (Fig. 1A2) and de-enhanced in the portal phase (Fig. 1A3). The mass had low signal intensity on T1-weighted MR images and high signal intensity on T2-weighted MR (Fig. 1B1 and 2). MR with perfusion-weighted imaging (MR-PWI) showed that the mass was heterogeneously enhanced in the arterial phase and hyper-enhanced in the portal phase (Fig. 1B3 and 4). In MR with diffusion-weighted images (MR-DWI), the mass was hyperintense with restricted diffusion (Fig. 1B5). Magnetic resonance cholangiopancreatography (MRCP) showed calculi in the main and left hepatic bile ducts as well as the gallbladder (Figs. 1C1 and 2). We reconstructed 3D images from the CT scans to illustrate the mass (Fig. 1D1) and the relationship between the mass and the hepatic vein, the portal vein, and their branches (Fig. 1D2). Surgery was performed in October 2016. Intraoperative ultrasound showed an 8 × 8 cm mass in segment VIII of the liver, and invasion of the diaphragm could be seen. The mass was observed to jostle against the right and middle hepatic veins. The edge of the mass was unclear. The patient underwent resection of liver segment VIII, regional lymphadenectomy and resection of lesions on the diaphragm. Next, we performed cholecystectomy and exploratory surgery of the common bile duct. Macroscopically, sporadic lesions surrounding the mass were seen. Histopathological examination showed hepatocellular cholangiocarcinoma with diffusion and infiltration of thrombi in some lymph vessels (Fig. 2). IHC staining showed EMA (+), CK19 (+), hepatocyte (−), Glypican-3 (−), Arginasel (−), AFP (−), CD34 (−), and Ki-67 positivity of 20%. The tumor proportion scores (TPS) of the PD-L1 and PD-1 expression levels were both <1%. Whole-exome sequencing (WES) was applied to the tissue resected during surgery, and the data were used to determine the presence of non-synonymous mutations (NSMs), as well as the status of TMB, MSI, and dMMR by bioinformatics methods. The TMB was determined to be 18.46 mutations/Mb, and a total of 28 NSMs were detected in the whole genome, including 2 insertion–deletion mutations (indels) and 26 single-nucleotidevariants (SNVs). SNVs were detected in *MSH2*, *MSH6*, *NR-21*, and *MONO-27*, suggesting dMMR, and MSI-H. This patient harboured clinically actionable mutation in *KIT*, *NRAS*, *TP53*, *MET*, *PDGFR*.

**Figure 3:**
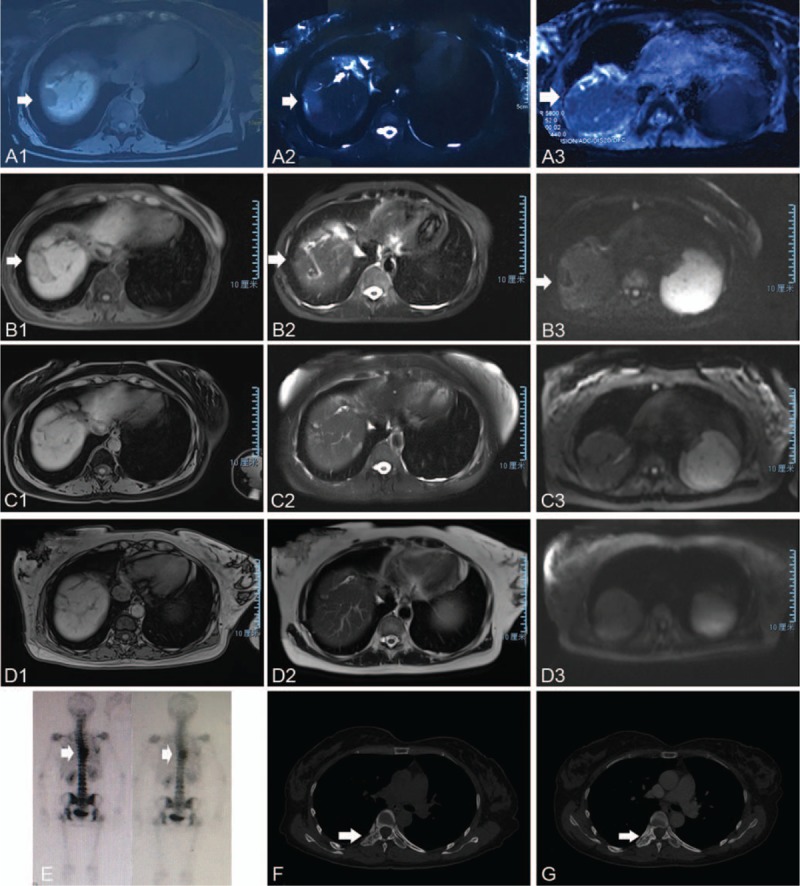
Postoperative radiological examination of the reported case. From (A) to (D), abdominal MRI scanning showed the lesions in the liver was becoming smaller and lessen at time points March 2017, June 2017, May 2018 and January 2019. Figures 3A1, 3B1, 3C1, 3D1 were the T1-weighted magnetic resonance (MR) image; Figures 3A2, 3B2, 3C2, 3D2 were the T2-weighted MR image; Figures 3A3 3B3 3C3 3D3 were the MR with diffusion-weighted images. The white arrow heads direct the liver lesion. (E, F) Emission Computed Tomography (ECT) scanning and bone window of chest routine scan showed metastases in the right thoracic vertebral pedicle at April 2017. (G) T-spine Routine Scan showed the metastases in the right thoracic vertebral pedicle were stable at January 2018. The white arrow heads direct the metastases.

**Figure 4:**
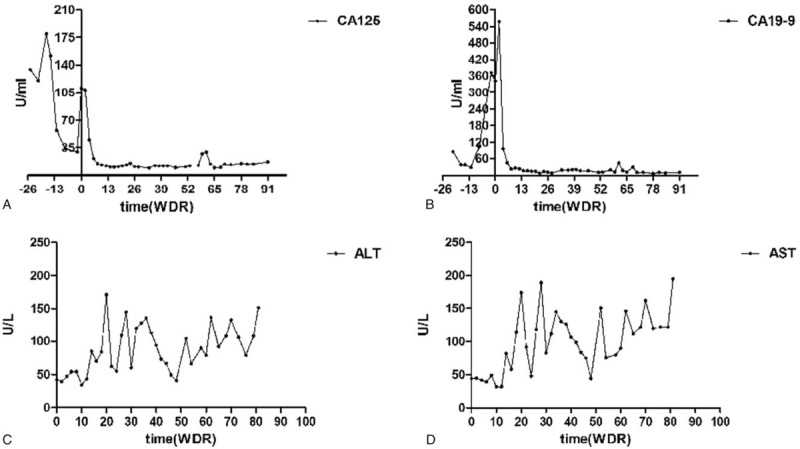
Preoperative and postoperative blood test results. (A) carbohydrate antigen 125 (CA125) level, (B) carbohydrate antigen 19-9 (CA19-9), (C, D) Alanine aminotransferase (ALT), aspartate aminotransferase (AST).

The postoperative course was uneventful. On the 7th postoperative day the patient had recovered and was discharged. The patient received 2 courses of chemotherapy (intravenous cisplatin 25 mg/m^2^ per day from day 1 to day 3, and oral xeloda 1000 mg/m^2^ per day from day 1 to day 14) and 1 course of radiotherapy after resection. Unfortunately, the patient developed recurrent lesions at 5 months after surgery. T1-weighted MR images showed that some masses were located in the right lobe of the liver and the largest 1 was 3.1 × 2.1 cm in size. T2-weighted MR images showed that the signal intensity of the mass was slight high; and MR-DWI showed that the masses were hyperintense with restricted diffusion (Fig. 3A). Percutaneous microwave ablation was performed in March 2017. Twelve days after percutaneous microwave ablation, the patient received the first cycle of nivolumab treatment (2 mg/kg). After 2 weeks, she received the second cycle of nivolumab and took lenvatinib (8 mg/day) simultaneously. During the subsequent maintenance phase, the patient received 3 mg/kg of nivolumab every 2 weeks plus lenvatinib at a dose of 10 mg/day. This combined treatment was continued for twenty months. After the treatment was initiated, follow-up MRIs performed in the 2nd month (Fig. 3B), 13th month (Fig. 3C), and 21st month (Fig. 3D) revealed that the lesions in the liver were becoming smaller and almost disappeared. To our surprise, during the second cycle of treatment, Emission Computed Tomography (ECT) (Fig. 3E) and CT (Fig. 3F) showed that CCA had expanded to the right thoracic vertebral pedicle (T7–8). However, CT showed that the metastases in the right thoracic vertebral pedicle were stable by the 9th month after therapy with nivolumab plus lenvatinib (Fig. 3G). The levels of tumor biomarkers decreased and became normal after the 4th cycle of treatment (Fig. 4A and B). MRI and CT showed that the patient achieved a partial response (PR) according to the standard RECIST 1.1 criteria. Unfortunately, the patient experienced treatment-related adverse events in the form of liver damage, hypertension, asymptomatic hypothyroidism, which were attributed to lenvatinib and determined to be of grade 2 according to the standard CTCAE5.0 criteria. After 1 cycle of combined treatment, serum alanine aminotransferase and aspartate aminotransferase levels were elevated (Fig. 4C and D), after which glycyrrhizinate and glutathione were administered. The drug-induced hypertension after 5 cycles was maintained in the normal range by taking a calciumchannel blocker (amlodipine 7.5 mg daily). After eight cycles, the patient was diagnosed with asymptomatic hypothyroidism, and 50 μg of levothyroxine was administered. The other adverse events to lenvatinib were epistaxis, hypoleukemia and fatigue, on which no management was performed.

To date, the tumor has regressed without recurrence. Final evaluation of treatment efficacy demonstrated a complete response.

## 3 Discussion

Cholangiocarcinoma is categorized according to its anatomical location as intrahepatic (iCCA), perihilar (pCCA), or distal (dCCA). Cholangiocarcinoma is an aggressive tumor with a dismal prognosis that poses significant therapeutic challenges. Hence, the development of novel treatment strategies is urgent. Surgery is the mainstay treatment option for all 3 disease subtypes, but only a subset of patients (approximately 35%) with early stage disease is suited for surgical resection with curative intent.^[22]^ For iCCA, surgical resection is associated with median disease-free survival (DFS) of 12 to 36 months, as reported in various patient series.^[23,24]^ Liver transplantation has conventionally been considered a contraindication for iCCA surgery, owing to a high risk of recurrence and poor survival outcomes.^[25,26]^ Locoregional therapies such as transcatheter arterial chemoembolization (TACE), radioembolization, or external-beam radiation therapy (EBRT) are a reasonable treatment approach for patients with advanced-stage iCCA who are not candidates for surgical resection. For patients with advanced-stage cholangiocarcinoma that is not suitable for surgical or locoregional options, the combination of cisplatin and gemcitabine constitutes the current first-line cytotoxic chemotherapy. Valle, et al reported that gemcitabine plus cisplatin therapy has a median overall survival of 11.7 months, vs 8.1 months with gemcitabine alone.^[27]^ More recently, molecularly targeted therapies are increasingly being investigated in early phase clinical trials in cholangiocarcinoma. Treatment options in phase II studies include receptor-tyrosine-kinase inhibitors such as NVPBGJ398, erdafitinib, and ponatinib, ALK and ROS1 inhibitors such as ceritinib and entrectinib, as well as the MEK inhibitor selumetinib.

Cancers utilize several mechanisms of immune escape to restrict or evade antitumor immune responses. These include loss of MHC expression, expression of immune-checkpoint proteins such as programmed cell death protein 1 (PD-1) and cytotoxic T-lymphocyte-associated antigen 4 (CTLA-4), as well as the regulation of the local tumor microenvironment to produce an immunosuppressive biochemical milieu. Recently the results of a phase I/II study aiming to reinvigorate the immune response to evaluate the efficacy and safety of immune-checkpoint inhibitors in cholangiocarcinoma were reported.^[28]^ PD-1, also known as CD279, is a co-inhibitory cell surface receptor that abrogates antitumor immune responses and promotes tumor immune escape from cytotoxic T cells during carcinogenesis.^[29]^ Therefore, blockade of the PD-1/PD-L1 pathway by interfering with the binding between PD-1 and its ligands is a potential strategy for cancer therapy. Mismatch repair (MMR) deficiency has also been demonstrated as an important predictive biomarker for immunotherapy. During normal DNA replication with proficient MMR (pMMR), small DNA mismatch errors are initially detected and corrected by the DNA MMR pathway. Deficiency in the DNA MMR pathway due to qualitative or quantitative abnormalities of the key proteins *MLH1*, *MSH2*, *MSH6*, and *PMS2* leads to accelerated accumulation of genetic errors (i.e., mutations) at microsatellites, leading to diffuse high levels of microsatellite instability (MSI-H). MMR deficiency in carcinoma has been shown to be a predictor of increased response to treatment with immune-checkpoint inhibitors.^[30]^ Resent studies demonstrate that dMMR status is predictive of a eutherapeutic effect of anti-PD-1-directed treatments in all types of cancer patients, regardless of the primary site.^[31]^ The tumor mutation burden (TMB) is another emerging biomarker that is associated with a greater likelihood of a response to immunotherapy.^[32]^ Increased TMB may produce neoantigens, whose recognition leads to lymphocyte infiltration in the tumor, which appears to be pivotal for the activity of checkpoint inhibitor immunotherapies that rely on PD-1, PD-L1or CTLA-4 blockade.^[13,33]^

Various antibodies against PD-1 and its ligands have been developed as biologicals and are currently being tested in clinical trials with liver cancer patients (Table 1). These antibodies include mAbs against PD-1 and PD-L1 fusion protein.

**Table 1:**
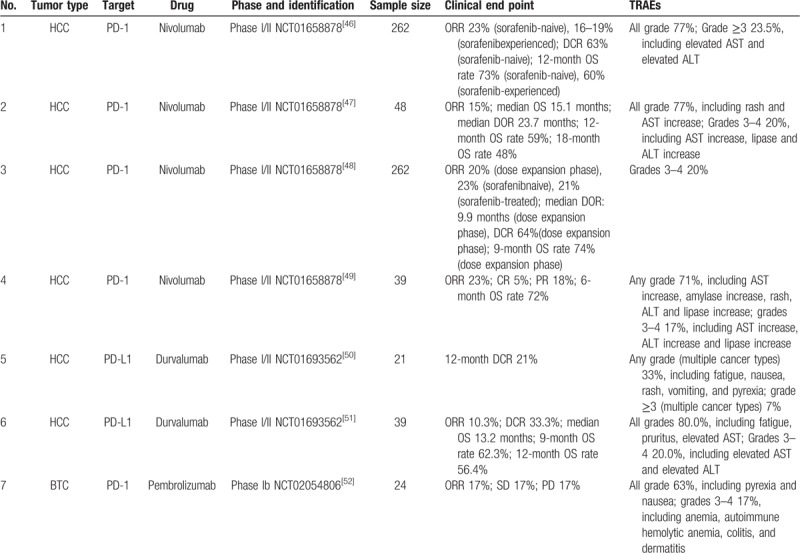
The key reported clinical trials of of PD-1/PD-L inhibitors in patients with hepatocellular carcinoma and biliary tract cancer.

At present, the clinical data on immunotherapy in cholangiocarcinoma is limited. However, numerous clinical trials are being conducted to investigate the effects of immunotherapy in biliary tract cancer (BTC). KEYNOTE-028 (NCT02054806), the most mature of these efforts, explored the effect of pembrolizumab in patients with BTC. Data from this study were recently published by Bang et al.^[9]^ In KEYNOTE-028, the overall response rate (ORR) was 17% and the disease control rate (DCR) was 34% with pembrolizumab monotherapy. The median progression-free survival (PFS) was 1.9 months and the median overall survival (OS) was 9.7 months. However, only 24 patients were enrolled in the study (20 with cholangiocarcinoma, 4 with gallbladder carcinoma) and all patients were preselected for ≥1% tumoral PD-L1 expression. The promising efficacy and safety of pembrolizumab in the KEYNOTE-028 phase Ib study prompted the enrollment of a successor cohort of 100 biliary cancer patients in the ongoing KEYNOTE-158 trial (NCT02628067). Furthermore, the PD-L1 inhibitor durvalumab is being tested as standalone immunotherapy in cohorts of patients affected by esophageal cancer or (NCT01938612).^[34]^ Phase II clinical trials (NCT02923934 and NCT02829918) of nivolumab as PD-1 immune checkpoint inhibitor for BTCs are in preparation. Several other studies of immune checkpoint inhibitors are now ongoing, including monotherapy trials and combinations with other drugs, including targeted drugs, chemotherapy, and other immunotherapies (Table 2).

**Table 2:**
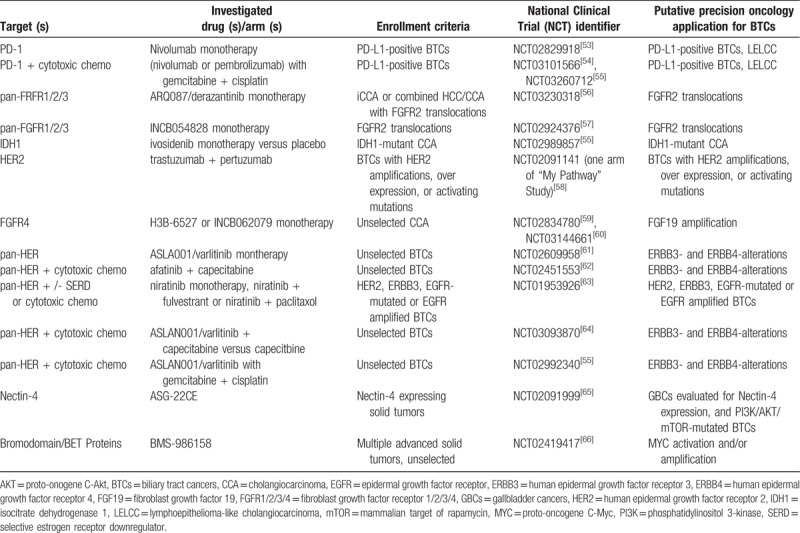
Highlighted ongoing clinical trials evaluating biliary tract cancers.

Here, we discuss a single case by highlighting the usage of the anti-PD-1 drug nivolumab in combination with the receptor tyrosine kinase inhibitor lenvatinib in a 40-year-old female with recurrent and metastatic iCCA after resection. This tumor showed deficiency in the mismatch repair (MMR) pathway and subsequent accumulation of replication errors with unstable abnormalities in short sequences of nucleotide (MSI-H). Furthermore, the tumor mutation burden (TMB) was very high, while PD-1 and PD-L1 expression was <1%. Based on the results of clinical studies, the U.S. FDA approved nivolumab for the treatment of patients with metastatic colorectal cancer with dMMR or MSI-H.^[35]^ Between March 12, 2014, and March 16, 2016, 74 patients were treated with nivolumab in the CheckMate 142 trial, for which Overman et al reported an overall response in 34%, or 25 patients (95% CI 23.2–45.7), including a complete response in 7 (9%). Disease control (≥12 weeks) was noted in 51 patients (69%, 95% CI 57–79). Median PFS was 6.6 months (95% CI 3.0-not estimable[NE]) and OS at 12 months was 44% (95% CI 19.6-NE). Both cholangiocarcinoma and colorectal cancer are types of adenocarcinoma.

According to the results of this case and the CheckMate 142 trial, nivolumab may provide promising and durable responses with prolonged survival relative to the anticipated median survival in patients with dMMR/MSI-H metastatic cholangiocarcinoma. Furthermore, FDA approved nivolumab for the treatment of HCC patients following prior sorafenib administration, regardless of the etiology of HCC or tumor expression of PD-L1.^[36]^ In the CheckMate-040 study, 154 patients with HCC who were intolerant to sorafenib or who progressed on sorafenib were enrolled to evaluate the efficacy of nivolumab. Base on the study results, the overall response rate (ORR) was 14.3%, with 1.9% complete response (CR), and 12.3% partial response (PR). Among those who responded to nivolumab, 91% had a response duration ≥ 6 months, and 55% achieved ≥ 12 months.

Currently, lenvatinib is FDA-approved for the treatment of patients with locally recurrent or progressive, metastatic, radioactive iodine (RAI)-refractory differentiated thyroid cancer (DTC). Its antitumor activity is attributed to its antiangiogenic properties and direct antitumor effects. In a phase II study in advanced RAI-refractory DTC, the median progression-free survival (PFS) of lenvatinib-treated patient was 12.6 months, with a 50% response rate (RR). In a phase III trial in RAI-refractory DTC, patients treated with lenvatinib achieved a 65% overall RR, with a median PFS of 18.3 months.^[37]^ The concept of targeted therapies has emerged as a promising approach for the treatment of HCC.^[38,39]^ New trials have been designed with the aim of evaluating the efficacy of lenvatinib as a targeted therapy. In 1 such study, 46 patients with advanced disease and Child Pugh A liver function status were enrolled to analyze the safety and efficacy of lenvatinib in a phase 1/2 open-label study. The initial treatment dose of lenvatinib was 2 mg daily (28-d cycles) until disease progression or development of unmanageable toxicities occurred. The median time to tumor progression (TTP) was 12.8 months (95%CI: 7.23–14.7), and the median overall survival was 18.7 months (95%CI: 12.8–25.1).^[18]^ The first-line treatments sunitinib, linifanib, and brivanib have all failed the phase III clinical trials. However, lenvatinib has passed the phase III clinical trial. A phase III study compared the efficacy of lenvatinib vs sorafenib as the first-line treatment for unresectable HCC. In this open-label study, 954 subjects with advanced HCC corresponding to Barcelona Clinic Liver Cancer Group stage B or C, and hepatic cirrhosis of Child-Pugh grade A were enrolled and randomized to receive either lenvatinib or sorafenib as first-line therapy. The results showed that lenvatinib had significant advantages in PFS, TTP, and ORR. The investigators concluded that lenvatinib demonstrated non-inferiority to sorafenib in overall survival.^[40]^ Despite such encouraging data, the efficacy of lenvatinib as a second-line treatment for patients with metastatic iCCA remains unclear. Only 1 phase II clinical study investigated the use of lenvatinib in biliary tract adenocarcinoma that failed to respond to gemcitabine-based therapy (NCT02579616). The exact mechanism governing the response to lenvatinib needs to be further clarified.

Immune-related adverse events (irAEs), which are induced by immune checkpoint inhibitors, can affect various organ systems. The most common immune-related adverse events of all grades caused by nivolumab involve fatigue, pruritus, and rash, followed by diarrhea, hypothyroidism, pneumonitis, autoimmune hepatitis, and nephritis.^[39]^ Hypophysitis occurred with low incidence rates, and was reported in lung cancer. ^[41–43]^ Other adverse events such as fever, hypoleukemia, and hydrothorax may also occur with nivolumab treatment.^[44]^ This patient experienced hypertension and epistaxis which are common adverse events of lenvatinib. One study reported that the incidence of all grades of irAEs was dose-dependent. ^[41]^ The incidence of irAEs during treatment with 1 mg/kg nivolumab was 58.08% (95% CI, 34.05–78.81), while it was 70.00% in patients administered 3 mg/kg nivolumab (95%CI, 21.76–95.14). The propensity of patients to withdraw or resume nivolumab after encountering irAEs depends on the type of adverse event.

A limitation of this study is that the patient received lenvatinib at a dose of 10 mg/day during the subsequent maintenance phase, which was not the recommended lenvatinib regimen. The expression of PD-L1 may be affected by both temporal fluctuations and intratumoral heterogeneity, so that low PD-1 and PD-L1 expression cannot completely predict whether a patient will benefit from immunotherapy. In tumors with dMMR, a high TMB and MSI-H is associated with a favorable response to immune checkpoint inhibitors.^[45]^ However, a recent study found that a high TMB, MSI-H, and PD-L1 expression also cannot completely predict whether patients could benefit from combination immunotherapy.^[1]^ A lack of the knowledge surrounding the underlying mechanism through which this patient benefitted from immunotherapy combined with lenvatinib is another limitation of the study.

To our best of knowledge, this is the first report of the use of nivolumab plus lenvatinib to successfully treat recurrent, progressive, metastatic cholangiocarcinoma. Positive dMMR/MSI-H and TMB-H in cholangiocarcinoma, as well as the suppression of tumor angiogenesis may provide mechanistic support for this treatment. Prospective studies are needed to validate the therapeutic efficacy and safety of nivolumab and lenvatinib in cholangiocarcinoma.

## Author contributions

**Conceptualization:** Weixun Chen, Ze-yang Ding.

**Data curation:** Weixun Chen.

**Formal analysis:** Weixun Chen.

**Funding acquisition:** Ze-yang Ding.

**Methodology:** Weixun Chen, Peng Zhu, Bi-xiang Zhang, Ze-yang Ding.

**Resources:** Weixun Chen, Gan-xun Li, Zheng-nan Hu, Peng Zhu, Bi-xiang Zhang, Ze-yang Ding.

**Software:** Weixun Chen.

**Writing – original draft:** Weixun Chen.

**Writing – review & editing:** Ze-yang Ding.
